# Designing an Online Tutorial on Persistent Physical Symptoms: How Can We Deliver Teaching on a Complex but Vital Topic Within a Busy Medical Student Curriculum?

**DOI:** 10.1192/bjo.2025.10304

**Published:** 2025-06-20

**Authors:** Jasmine Schulkind, Jahnavi Acharya, Joanne Davies, Kate Seddon

**Affiliations:** 1 Avon & Wiltshire Mental Health Partnership NHS Trust, Bristol, United Kingdom; 2 University Hospitals Bristol and Weston NHS Foundation Trust, Bristol, United Kingdom

## Abstract

**Aims:** Improve the knowledge and confidence of Year 4 Bristol medical students in assessing and managing patients presenting with persistent physical symptoms (PPS) through the delivery of an online teaching package.

**Methods:** We assessed outcomes using PDSA cycles.

Plan – We first identified the problem: Bristol medical students report low confidence and knowledge in assessing and managing patients presenting with PPS.

We then designed an intervention in the form of an online teaching package delivered to all Year 4 Bristol medical students during their psychiatry placement. The teaching package included:

Pre-module knowledge quiz.

2 recorded Powerpoint lectures covering aetiology, pathophysiology, assessment, management and prognosis for PPS.

2 × 20–30 minute videos. With support from Bristol University, we filmed 2 mock consultations with Consultants working at the local specialist neuropsychiatric unit assessing patients (actors) presenting with PPS.

Clinical knowledge quiz based on videos.

Chronic pain lived experience video followed by knowledge quiz.

PDSA 1 – piloted the teaching package to a cohort of 20 students. Collected written feedback and subsequently edited recorded lectures and questionnaires.

PDSA 2 – rolled out to all 4th year medical students in Severn Deanery from September 2023. Collected pre- and post-course questionnaires (69 students September 2023–January 2024) to assess change in knowledge/confidence.

PDSA 3 – Adapted the intervention based on written feedback. Added in a chronic pain lived experience video and knowledge questionnaire.

**Results:** We compared pre- and post-module quantitative and qualitative feedback. Mean questionnaire scores from PDSA cycle 2 (69 students) demonstrated significant improvement in a) confidence in history taking (3.4 to 7.0 on Likert scale) and b) confidence in explaining a diagnosis of PPS (5.2 to 7.3 Likert scale) following the intervention. On average students scored 7.9 (Likert scale 0: not at all useful to 10: extremely useful) to the question ‘How useful do you think this module will be for your future clinical practice?’

**Conclusion:** Despite the high burden of PPS within both primary and secondary care, the topic is poorly covered within medical student curriculum nationally. Bristol medical students report low confidence and knowledge in assessing and managing patients presenting with PPS.

Adding in content to an already busy 6-week medical student psychiatric curriculum is challenging. Our intervention has demonstrated that an interactive e-learning tutorial involving recorded lectures, clinical videos and knowledge quizzes is an effective method to improve students’ knowledge and confidence for this important topic.

